# The Negative Impacts of Sarcopenia on Primary Total Knee Arthroplasty under the Enhanced Recovery after Surgery Protocol

**DOI:** 10.1111/os.14053

**Published:** 2024-03-31

**Authors:** Shengliang Zhou, Lan Li, Shuai Li, Haibo Si, Limin Wu, Bin Shen

**Affiliations:** ^1^ Department of Orthopedic Surgery and Orthopedic Research Institute, West China Hospital Sichuan University Chengdu China; ^2^ Department of Endocrinology and Metabolism and Key Lab of Transplant Engineering and Immunology, NHFPC, West China Hospital Sichuan University Chengdu China

**Keywords:** Complications, Enhanced recovery after surgery, Forgotten joint score, Sarcopenia, Total knee arthroplasty

## Abstract

**Objective:**

Sarcopenia, as an emerging public health concern, has been associated with postoperative adverse outcomes in various surgical procedures. However, the evidence regarding the impacts of sarcopenia on total knee arthroplasty (TKA) remained limited. This study aimed to assess the impacts of sarcopenia on primary TKA based on the enhanced recovery after surgery (ERAS) protocol.

**Methods:**

This retrospective study included 291 patients who received unilateral TKA from October 2017 to May 2018 in our institution. Sarcopenia was diagnosed based on the algorithm of Asian Working Group for Sarcopenia 2019. The handgrip strength was measured using a handheld dynamometer and the muscle mass was estimated by a previously validated anthropometric equation. Patients were classified into sarcopenia group and non‐sarcopenia group. The outcomes included complications, postoperative length of stay (LOS), total hospitalization cost, operative time, total estimated blood loss, blood transfusion rate, and the 12‐item forgotten joint score (FJS‐12) at the follow‐up. The propensity score matching (PSM) was used to adjust confounding factors. We compared continuous variables using Student's *t*‐test and the Wilcoxon Mann–Whitney *U* test for normal and non‐normal distributions, respectively, and categorical variables with chi‐square tests.

**Results:**

Of the 291 patients, 58 (19.9%) patients were identified as having sarcopenia. After PSM, each group matched 42 patients. All matched patients were followed‐up at least 5 years. Patients with sarcopenia had higher rates of surgical complications compared to the non‐sarcopenia group (*p* = 0.019), and no significant difference was observed in 30‐day readmission, and periprosthetic joint infection. The sarcopenia group had significantly longer LOS (*p* = 0.038), higher total hospitalization (*p* = 0.015) than the non‐sarcopenia group. For the FJS‐12 scores at follow‐up, patients with sarcopenia had significantly higher scores than the non‐sarcopenia group (*p* = 0.024).

**Conclusion:**

Our findings indicated sarcopenia may be a risk factor for postoperative complications, prolonged LOS, increased hospitalization cost and reduced patient satisfaction.

## Graphical abstract

This study included 291 patients who received unilateral total knee arthroplasty in our institution. The handgrip strength and muscle mass were used to assess the sarcopenia and 58 patients with sarcopenia were identified. After propensity score matching, 84 patients were matched. All matched patients were followed‐up at least 5 years. At last, we found that sarcopenia was a risk factor for postoperative complications, prolonged length of stay, increased hospitalization costs and reduced patient satisfaction.

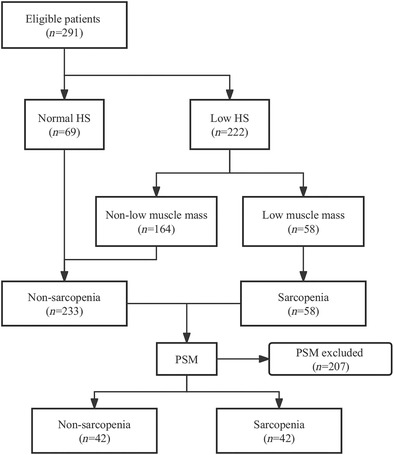

## Introduction

Sarcopenia, an age‐related geriatric syndrome, is defined as low muscle mass plus low muscle strength or low physical performance according to the Asian Working Group for Sarcopenia (AWGS) 2019.^1^ Sarcopenia has been reported to be associated with an increased risk of a range of adverse outcomes, such as falls, frailty, and mortality.^2^, ^3^, ^4^ The prevalence of sarcopenia ranges from 10% to 27% worldwide due to the use of various diagnostic methods, and patients with osteoarthritis (OA) are at an increased risk for developing sarcopenia.^5^, ^6^ Recently, there has been a growing emphasis on exploring the impact of sarcopenia on postoperative complications. Several studies have indicated that sarcopenia serves as a predictor of complications and poor prognosis following various surgical procedures, such as gastrointestinal surgery, head and neck surgery, and liver transplantation.^7^, ^8^, ^9^ In the context of total knee arthroplasty (TKA), previous studies have reported that sarcopenia is associated with increased risk of prolonged length of stay, falls, reoperation, and prosthetic joint infection.^10^, ^11^, ^12^ Nevertheless, the impact of sarcopenia on total joint arthroplasty outcomes remains ambiguous, largely attributable to limited follow‐up durations and the scarcity of studies assessing the impact of sarcopenia under the enhanced recovery after surgery (ERAS) protocols.

Therefore, to fill this gap, we conducted this retrospective cohort study to evaluate the impacts of sarcopenia on postoperative outcomes in TKA patients under the ERAS protocol. The aims of this study were to help determine whether sarcopenia: (i) affects the postoperative complications; and (ii) affects the LOS, total hospitalization cost, and blood loss.

## Methods

### Study Design

We retrospectively collected the information of all consecutive patients who underwent primary TKA from October 2017 to May 2018 at our institution. The relevant in‐hospital information of patients was collected by checking the medical records of the Hospital Information System (HIS) at our institution. The inclusion criteria were: (i) patients received primary TKA; (ii) patients were diagnosed with knee OA; (iii) patients were measured the handgrip strength (HS); and (iv) patients with complete medical records and laboratory data. Patients who underwent simultaneous or staged bilateral TKA were excluded. All included patients were classified into two groups: the sarcopenia group and the non‐sarcopenia group.

**FIGURE 1:**
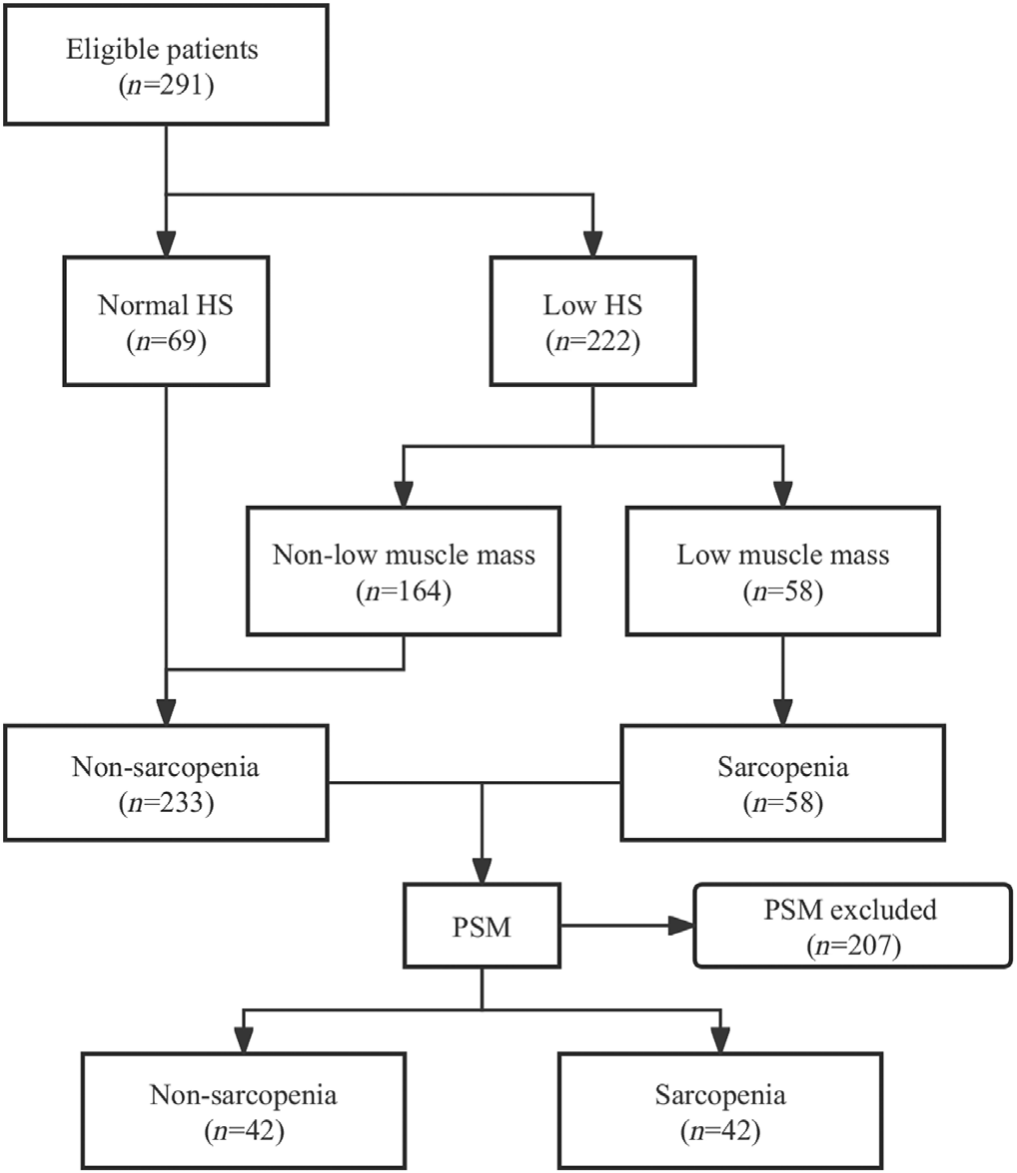
Flowchart of patient selection. HS, handgrip strength; LMM, low muscle mass; PSM, propensity score matching.

According to the inclusion and exclusion criteria, a total of 291 patients were eligible. Of the 291 patients, 58 (19.9%) were diagnosed with sarcopenia, and 233 (80.1%) were not diagnosed with sarcopenia. We subsequently conducted 1:1 propensity score matching (PSM) to match patients with sarcopenia and without sarcopenia and each group matched with 42 patients (Figure 1). All matched patients finished at least 5 years of follow‐up. The comprehensive methodology utilized for the PSM was elaborated in the Statistical Analysis section.

This retrospective cohort study was performed with the approval of our institutional review board of the West China Hospital of Sichuan University (No. 201302007).

### ERAS Protocol

All patients received the same ERAS protocol. During the preoperative period, patients were provided the same preoperative education and physical exercise. Patients were encouraged to engage in bed exercises to enhance the strength of the quadriceps femoris muscles, improve the range of motion of the joint, and consume a high protein diet for nutritional support. Patients in the study received 200 mg of celecoxib (Celebrex, Pfizer, New York) twice a day, starting 2 days prior to the operation and continuing for 2 weeks after surgery. All primary TKA surgeries were conducted *via* the standard mid‐vast approach under general anesthesia. Prior to general anesthesia, all patients underwent a saphenous nerve block using 30 mL of 0.33% ropivacaine, guided by ultrasound, in the operating room. Additionally, all patients received intravenous tranexamic acid (20 mg/kg) 10 min before surgery, followed by 1 g of tranexamic acid intravenously at 3‐ and 6‐h post‐surgery. A standardized thromboembolism prophylaxis protocol was administered to all patients after surgery. It was recommended that patients sit at the bedside and perform straight leg raising exercises starting on the evening after surgery.

### Sarcopenia Assessment

Based on the AWGS 2019, we assessed sarcopenia using muscle strength and appendicular skeletal muscle mass (ASM).^1^ The muscle strength was assessed by handgrip strength (HS), which was measured using a handheld dynamometer with a precision of 0.1 kg. Both hands were assessed with the participants in a seated position. The wrist was maintained in a neutral position, and the elbow was flexed at a 110° angle. A low HS was defined as having a maximum HS of less than 28 kg in men and less than 18 kg in women.^1^ The ASM was calculated using a previously validated equation in a Chinese population, which has shown good consistency with dual‐energy X‐ray absorptiometry (DXA) (adjusted *R* ^2^ = 0.90, the standard error of estimate = 1.63 kg).^13^ Specifically, the equation was as follows: ASM = 0.193 × weight (kg) + 0.107 × height (cm)—4.157 × sex—0.037 × age (years)—2.631.^13^ The age, sex, weight and height of all patients were recorded at admission. The men were set as 1 and the women were set as 2. According to previous studies, the calculated ASM was divided by the square of the height in meters, which was defined as the skeletal muscle mass index (SMI, SMI = ASM/height^2^).^14^, ^15^, ^16^ The definition of low muscle mass was based on the sex‐specific lowest 20% of SMI in the study population.^14^, ^17^, ^18^ In this study, patients were classified as having low muscle mass if they had a SMI ≤7.05 kg/m^2^ in men and SMI ≤5.55 kg/m^2^ in women. Patients with low HS and low muscle mass were diagnosed with sarcopenia.

### Outcomes Measures

The demographic data of all patients at admission including age, sex, ethnicity, surgical side, smoking status, drinking status, the American Society of Anesthesiologists (ASA) grade, hypertension, diabetes mellitus (DM), and osteoporosis were collected from the medical records. Preoperative hemoglobin (Hb), preoperative hematocrit (Hct) and preoperative albumin (Alb) were also collected.

#### Complications

Complications included medical complications, surgical complications, 30‐day readmission and PJI. Medical complications included cardiovascular (arrhythmia), gastrointestinal (postoperative nausea and vomiting, constipation), respiratory (pulmonary infection), genitourinary (urinary retention) and neurological (stroke) complications. Surgical complications included wound leakage, edema of the operated limbs, wound dehiscence, superficial wound infection and calf muscular vein thrombosis. Wound leakage was defined as postoperative wound leakage lasting for more than 72 h.^19^, ^20^ All complications were recorded during hospitalization and the follow‐up period. All matched patients were asked to return to the outpatient clinic for follow‐up. If they did not return, the complications information was collected via telephone or email.

#### LOS, Total Hospitalization Cost and Operative Time

LOS was defined as the number of days from surgery to discharge according to the medical records. The postoperative discharge criteria were as follows: effective pain control with oral analgesics; independent ability to transfer and walk alone for ≥60 m; and absence of signs such as redness, swelling, or bleeding at the surgical sites. The total hospitalization cost of each patient was collected from medical records and the unit was RMB. The operative time data were collected from the medical records.

#### Total Estimated Blood Loss

The total estimated blood loss was calculated according to previous studies,^21^, ^22^ taking into account factors such as sex, height, weight, and preoperative and postoperative Hb.

#### Blood Transfusion Rate and Maximal Hb, Hct, and Alb Reductions

Blood transfusion rates and postoperative Hb, Hct, and Alb levels were collected from the medical records. The maximal reductions in Hb, Hct, and Alb were defined as the differences between the preoperative values and the lowest postoperative values of Hb, Hct, and Alb during hospitalization.

#### 12‐Item Forgotten Joint Score

The 12‐item forgotten joint score (FJS‐12) is a patient‐reported outcome measure. It consists of 12 items to assess joint awareness during the activities of daily living.^23^ Each item uses a five‐point Likert scale with the following options: never, almost never, seldom, sometimes, and mostly. The raw total score was divided by the number of completed items, and then was multiplied by 25. The obtained score was subtracted from 100, and was ultimately converted into a scale ranging from 0 (the worst) to 100 (the best).^23^ Higher scores indicate better outcomes. The FJS‐12 score was assessed during the follow‐up period. If patients did not return to the outpatient clinic, the information was collected via telephone or email.

### Statistical Analysis

Continuous variables are presented as the means ± standard deviations (SDs) or medians (interquartile ranges). Student's *t*‐test and the Wilcoxon Mann‐Whiney *U* test were performed for normally and nonnormally distributed variables, respectively. Categorical variables are presented as numbers with percentages, and chi‐square tests were performed. To mitigate the impact of confounding variables on the comparison between sarcopenia and non‐sarcopenia groups, we employed PSM. Propensity scores were estimated using logistic regression, with sarcopenia as the dependent variable and the baseline characteristics with the *p*‐value <0.1, including age, ethnicity, hypertension, DM, osteoporosis, preoperative Hb, Hct, and Alb as covariates. We used nearest neighbor matching with a 1:1 ratio and a caliper width of 0.02 standard deviations of the logit of the propensity score. After matching, the characteristics of the two groups were similar. All statistical analyses in this study were conducted with SPSS version 26 (SPSS, Inc., Chicago, IL, USA). The level of statistical significance was set at *p* < 0.05 (two‐sided).

## Results

### Patient Characteristics

The baseline characteristics were presented in Table 1. Before matching, patients with sarcopenia were more likely to be older, and more likely to have hypertension, DM, and osteoporosis than patients in the non‐sarcopenia group. According to the preoperative blood test, patients with sarcopenia had lower preoperative Hb, Hct and Alb levels than patients in the non‐sarcopenia group. After matching, no significant differences were observed in any patient characteristic (Table 1).

**TABLE 1:** Baseline characteristics of included patients before and after matching

Variables	Before matching			After matching		
	Sarcopenia	Non‐sarcopenia	*p*‐value	Sarcopenia	Non‐sarcopenia	*p*‐value
*N*	58	233		42	42	
Age, years	71.81 ± 7.28	66.88 ± 7.22	<0.001	69.57 ± 6.73	70.95 ± 5.51	0.306
Sex			0.989			1
Male	10 (17.24)	40 (17.17)		8 (19.05)	8 (19.05)	
Female	48 (82.76)	193 (82.83)		34 (80.95)	34 (80.95)	
Ethnicity			0.013			0.137
Han	56 (96.55)	196 (84.12)		40 (95.24)	36 (85.71)	
Minority	2 (3.45)	37 (15.88)		2 (4.76)	6 (14.29)	
Surgical side			0.618			0.827
Right	31 (53.45)	133 (57.08)		21 (50)	22 (52.38)	
Left	27 (46.55)	100 (42.92)		21 (50)	20 (47.62)	
Drinking			0.773			0.724
Yes	5 (8.62)	23 (9.87)		5 (11.9)	4 (9.52)	
No	53 (91.38)	210 (90.13)		37 (88.10)	38 (90.48)	
Smoking			0.808			1
Yes	3 (5.17)	14 (6.01)		2 (4.76)	2 (4.76)	
No	55 (94.83)	219 (93.99)		40 (95.24)	40 (95.24)	
ASA			0.592			0.419
1	4 (6.9)	14 (6.14)		3 (7.14)	2 (5.00)	
2	34 (58.62)	153 (67.11)		26 (61.9)	20 (50)	
3	20 (34.48)	60 (26.32)		13 (30.95)	18 (45)	
4	0 (0)	1 (0.44)		0	0	
Hypertension			0.016			0.383
Yes	23 (39.66)	133 (57.33)		19 (45.24)	23 (54.76)	
No	35 (60.34)	99 (42.67)		23 (54.76)	19 (45.24)	
DM			0.02			0.355
Yes	21 (36.21)	50 (21.55)		12 (28.57)	16 (38.10)	
No	37 (63.79)	182 (78.45)		30 (71.43)	26 (61.90)	
Osteoporosis			0.001			0.178
Yes	15 (25.86)	23 (9.91)		7 (16.67)	3 (7.14)	
No	43 (74.14)	209 (90.09)		35 (83.33)	39 (92.86)	
Preoperative Hb, g/L	125.71 ± 11.69	133.01 ± 13.95	<0.001	129.38 ± 10.45	127.40 ± 13.72	0.459
Preoperative Hct	0.39 ± 0.03	0.41 ± 0.03	<0.001	0.40 ± 0.03	0.39 ± 0.03	0.383
Preoperative Alb, g/L	44.63 ± 2.69	46.06 ± 3.48	<0.001	45.21 ± 2.49	44.26 ± 2.99	0.117
Handgrip strength, kg	18.07 ± 3.87	23.75 ± 6.04	<0.001	18.30 ± 3.84	24.07 ± 7.01	<0.001
SMI, kg/m^2^	5.45 ± 0.62	6.75 ± 0.85	<0.001	5.49 ± 0.61	6.75 ± 0.83	<0.001

*Note*: Continuous variables are presented as the mean ± standard deviation, and categorical variables are expressed as numbers (percentages).

Abbreviations: Alb, albumin; ASA, American Society of Anesthesiologists (ASA) grade; DM, diabetes mellitus; Hb, hemoglobin; Hct, hematocrit; HS, handgrip strength; SMI, skeletal muscle mass index.

### Complications

There were 11 (26.19%) patients and 3 (7.14%) patients with surgical complications in the sarcopenia group and non‐sarcopenia group, respectively (*p* = 0.019) (Table 2). Wound leakage (*p* = 0.021) and edema of operated limbs (*p* = 0.048) significantly differed between the two groups. There were no significant differences in other surgical complications and medical complications. No patient reported 30‐day readmission or PJI in the follow‐up period (Table 2). Two typical cases were presented in the supplementary material.

**TABLE 2:** Postoperative complications according to sarcopenia and non‐sarcopenia groups

Outcomes	Sarcopenia	Non‐sarcopenia	*p*‐value
*N*	42	42	
Medical complications	4 (9.52)	5 (11.9)	0.724
Cardiovascular	1 (2.38)	1 (2.38)	1
Gastrointestinal	3 (7.14)	2 (4.76)	0.645
Respiratory	0	2 (4.76)	0.152
Genitourinary	0	1 (2.38)	0.314
Neurological	0	0	NA
Surgical complications	11 (26.19)	3 (7.14)	0.019
Wound leakage	5 (11.9)	0	0.021
Edema of operated limbs	6 (14.29)	1 (2.38)	0.048
Wound dehiscence	2 (4.76)	0	0.152
Superficial wound infection	0	1 (2.38)	0.314
Calf muscular vein thrombosis	1 (2.38)	1 (2.38)	1
30‐day readmission	0	0	NA
Periprosthetic joint infection	0	0	NA

*Note*: Data are expressed as numbers (percentages).

### LOS, Total Hospitalization Cost and Operative Time

Table 3 shows the comparisons of LOS, total hospitalization cost and operative time between sarcopenia and non‐sarcopenia groups. Compared with the non‐sarcopenia group, patients with sarcopenia had significantly longer LOS (3.28 ± 0.77 *vs* 3.69 ± 0.97 days, *p* = 0.038). In addition, the total hospitalization cost in the sarcopenia group was significantly higher than that in the non‐sarcopenia group (*p* = 0.015). No significant difference between the two groups in operative time.

**TABLE 3:** Clinical outcomes according to sarcopenia and non‐sarcopenia groups

Outcomes	Sarcopenia	Non‐sarcopenia	*p*‐value
*N*	42	42	
LOS (days)	3.69 ± 0.97	3.28 ± 0.77	0.038
Total hospitalization cost	51441.85 (49580.82–55023.14)	49213.31 (47790.2–51725.47)	0.015
Operative time (min)	85.71 ± 30.05	96.58 ± 32.52	0.117
Total estimated blood loss (mL)	592.76 ± 249.37	565.16 ± 304.64	0.651
Blood transfusion rate (*n* [%])	3 (7.14)	1 (2.38)	0.306
Maximal Hb reduction (g/L)	24 ± 9.98	18.76 ± 10.99	0.02
Maximal Hct reduction	0.07 ± 0.03	0.05 ± 0.03	0.012
Maximal Alb reduction (g/L)	8.04 ± 2.9	6.86 ± 3.23	0.083
FJS‐12	67.59 ± 9.81	72.47 ± 9.61	0.024

*Notes*: Continuous variables are presented as the mean ± standard deviation or median (interquartile range). Categorical variables are expressed as numbers (percentages).

Abbreviations: Alb, albumin; FJS‐12, 12‐item forgotten joint score; Hb, hemoglobin; Hct, hematocrit; LOS, postoperative length of stay.

### Total Estimated Blood Loss and Blood Transfusion Rate

The total estimated blood loss was similar between the sarcopenia and non‐sarcopenia groups (592.76 ± 249.37 *vs* 565.16 ± 304.64 mL). Three patients received blood transfusion in the sarcopenia group and one patient in the non‐sarcopenia group, with no significant difference between groups (*p* = 0.306) (Table 3).

### FJS‐12

In terms of FJS‐12 scores at follow‐up, the sarcopenia group had significantly lower scores than the non‐sarcopenia group (67.59 ± 9.81 *vs* 72.47 ± 9.61, *p* = 0.024) (Table 3).

## Discussion

### Summary of Results

We conducted this retrospective study using data from our institution to assess the impacts of sarcopenia on postoperative outcomes following primary TKA. The main findings of this study were that primary TKA patients with sarcopenia showed higher rates of postoperative surgical complications, longer LOS, higher total hospitalization costs, and lower FJS‐12 scores than patients without sarcopenia. We did not find any significant difference in the blood transfusion rate or medical complications.

### Postoperative Complications

Several studies have reported that sarcopenia is an independent negative prognostic factor for postoperative complications after various surgical procedures.^7^, ^24^, ^25^ Consistent findings were also observed for TJA. Babu *et al*. reported that the psoas‐lumbar vertebral index, a marker for central sarcopenia, was an independent predictor of PJI after TJA.^10^ Ardeljan *et al*. indicated that sarcopenic patients undergoing primary TKA had a higher risk of falls, reoperations, mechanical complications and PJI.^11^ He *et al*. reported that patients with sarcopenia who underwent TKA had increased postoperative complication rates, such as nausea, vomiting, edema of the operated limb and deep vein thrombosis, compared to the healthy control group.^12^ These results were generally in line with our findings. Compared with those in the non‐sarcopenia group, patients with sarcopenia had higher surgical complication rates after TKA in this study. This may be because sarcopenia is associated with a reduction in protein reserves, which can affect wound healing. However, we did not observe any PJI between the two groups. The association between sarcopenia and PJI should be further elucidated in future studies with larger sample sizes and longer follow‐up.

### LOS and Total Hospitalization Costs

Previous studies have yielded conflicting findings regarding the impact of sarcopenia on the LOS in patients undergoing TKA. Babu *et al*. reported that patients with sarcopenia had similar LOS compared to the non‐sarcopenia group.^10^ Another study also demonstrated that there was no significant difference in the LOS between sarcopenia patients and non‐sarcopenia patients.^12^ However, Bokshan *et al*. found sarcopenia was associated with a longer LOS and an increased risk of in‐hospital complications in patients who underwent thoracolumbar spine surgery.^26^ In addition, a retrospective matched control study showed patients with sarcopenia had an approximately 5% longer hospital stay compared to controls.^11^ Consistent with these studies, our study also identified a significant difference in the LOS between the sarcopenia and non‐sarcopenia groups. The prolonged LOS in the sarcopenia group may be attributed to the increased risk of in‐hospital complications. Moreover, prolonged hospital stays have been strongly associated with increased costs of TKA patients, according to prior studies.^27^, ^28^ Therefore, it was reasonable that the total hospitalization cost was higher in the sarcopenia group than in the non‐sarcopenia group in our study.

### Total Estimated Blood Loss and Blood Transfusion

In this study, we did not observe a statistically significant difference in total estimated blood loss and postoperative blood transfusion between the sarcopenia and non‐sarcopenia groups, which was inconsistent with previous studies. Hwang *et al*. examined 452 patients who underwent TKA and found low muscle mass was an independent risk factor for postoperative transfusion.^29^ In addition, He *et al*. demonstrated that the sarcopenia group had a higher blood transfusion rate than the non‐sarcopenia group in patients undergoing TKA.^12^ The discrepancies between our findings and previous studies could be attributed to the use of the ERAS protocol. The existing evidence supports the effectiveness of ERAS in decreasing postoperative transfusion rates following TKA.^30^, ^31^ In this study, surgery was performed under the minimally invasive concept to avoid unnecessary vascular damage. In addition, intravenous tranexamic acid was administered both pre‐ and post‐operatively. These measures may contribute to the reduction in intraoperative blood loss and postoperative transfusion rates among patients with sarcopenia.

### Forgotten Joint Score

Joint awareness is a relatively novel dimension of patient‐reported outcomes, which refers the ability to forget joint arthroplasty during daily living activities.^23^ Joint awareness can be assessed using the FJS‐12 scale.^32^, ^33^ The FJS‐12 has been translated into many languages and has shown good validity and reliability.^34^ Compared to other patient‐reported outcomes, the FJS‐12 scores have lower ceiling effects.^34^ However, few studies have evaluated the patient‐reported outcomes of sarcopenic patients who underwent primary TKA. We found that patients in the sarcopenia group had significantly lower FJS‐12 scores than patients in the non‐sarcopenia group. A lower FJS‐12 score indicated that sarcopenic patients may have worse postoperative knee function and satisfaction following TKA. Previous studies have indicated that sarcopenic patients with TKA had lower postoperative knee society scores^12^ and were more likely to experience postoperative walking disability.^35^ These results suggested that lower FJS‐12 scores in patients with sarcopenia were probable.

### Strengths and Limitations

The strength of this study was that it was the first study to investigate the impacts of sarcopenia on postoperative outcomes of primary TKA under the ERAS protocol. This study has the potential to increase the awareness of clinicians regarding the impact of sarcopenia on TKA, and facilitate further optimization of ERAS protocols for TKA in individuals with sarcopenia, thereby improving the safety and benefits of the perioperative period for this population. For patients identified with sarcopenia before TKA, it is imperative to enhance the ERAS protocol accordingly. This enhancement should encompass strategies such as preoperative nutritional supplementation and muscle resistance training, alongside postoperative rehabilitation tailored to the individual's needs.^36^ These measures are crucial for mitigating the adverse effects of sarcopenia on surgical outcomes and facilitating a more robust recovery process.

However, there are also several limitations that should be noted. First, this was a single‐center study with a limited sample size. Further prospective studies with larger sample sizes are needed to corroborate the findings. Second, we recorded the FJS‐12 scores only at 5 years post‐surgery, without presenting temporal trends in FJS‐12 scores between individuals with and without sarcopenia. Third, muscle mass was assessed using a previously published equation and was not assessed by DXA. Although the equation has been validated in many studies and has demonstrated good consistency with DXA in the Chinese population, muscle mass was not assessed by objective measures.^13^, ^14^, ^15^, ^16^

## Conclusion

In conclusion, our findings indicated that sarcopenia was a risk factor for postoperative surgical complications, prolonged LOS, increased total hospitalization cost, and decreased FJS‐12 scores in patients undergoing primary TKA. We did not observe a significant difference in the postoperative transfusion rates between the sarcopenia and non‐sarcopenia groups. These findings emphasize the need to identify sarcopenia preoperatively to optimize postoperative recovery and enhance patient satisfaction. Further studies with larger sample sizes are warranted to validate in the future.

## Conflict of Interest Statement

All authors declare that they have no conflict of interest with other people or organizations that could inappropriately influence this work.

## Author Contributions

Study design and manuscript writing: S.Z., S.L. and B.S.; data collecting: S.Z., and L.L.; statistical analysis: S.Z., and H.S.; data checking: L.W. and B.S. All authors have read and agreed to the published version of the manuscript.

## Funding Information

This study was supported through grants from the National Natural Science Foundation of China (Nos. 82272561 and 81974347), the Foundation of the Science & Technology Department of Sichuan Province (Program No. 2022YFS0050).

## Supporting information


**Data S1.** Supporting Information.
