# Supplementary material for: The Negative Impacts of Sarcopenia on Primary Total Knee Arthroplasty under the Enhanced Recovery after Surgery Protocol
Source: Orthop Surg. 2024 Mar 31;16(5):1160–7. doi: 10.1111/os.14053 (PMC11062878; doi:10.1111/os.14053)
Supplement: Supplementary file 1 — Data S1. Supporting Information. [file OS-16-1160-s001.docx]

**Cases one**

A 62-year-old woman underwent a left total knee arthroplasty at our institution. With a height of 1.5 m and weight of 45 kg, the appendicular skeletal muscle mass (ASM) was calculated at 11.46 kg, resulting in a skeletal muscle mass index of 5.10 kg/m^2^. The grip strength was measured as 17.5 kg. Based on diagnostic criteria for sarcopenia, she was classified as sarcopenia. Postoperatively, wound leakage and swelling occurred on the first day. The estimated total blood loss was 650 mL. She was discharged on the third postoperative day, with a total cost of 58552.36 RMB. At the five-year follow-up, the FJS-12 score was 52 (Figure A).

**Cases two**

A 72-year-old woman underwent a left total knee arthroplasty at our institution. With a height of 1.5 m and weight of 59 kg, the appendicular skeletal muscle mass (ASM) was calculated at 13.68 kg, resulting in a skeletal muscle mass index of 6.08 kg/m^2^. The grip strength was measured as 19.8 kg. Therefore, she was classified as non-sarcopenia. No complication occurred postoperatively. The estimated total blood loss was 624 mL. She was discharged on the third postoperative day, with a total cost of 46625.06 RMB. At the five-year follow-up, the FJS-12 score was 75 (Figure B).


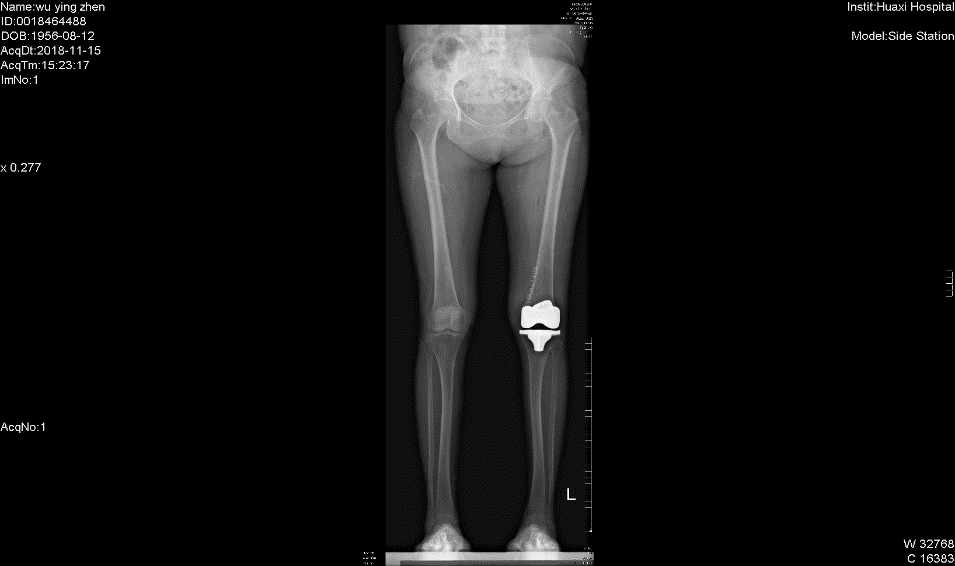

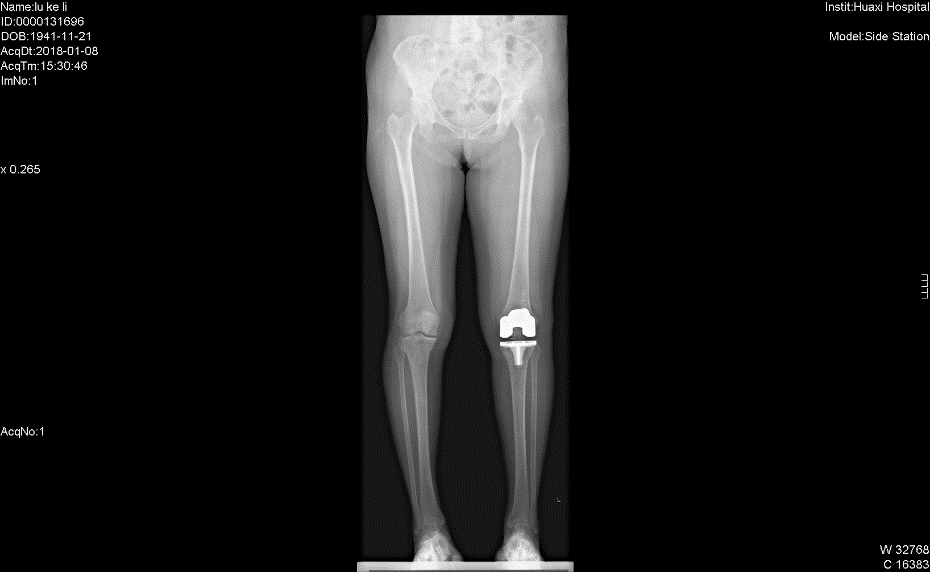


1. (B)

**Figure legend** The X-ray images of the full-length lower limbs after total knee arthroplasty. (A) Case one. (B) Case two.
